# Supplementary material for: An open dataset of Plasmodium falciparum genome variation in 7,000 worldwide samples
Source: Wellcome Open Res. 2021 Jul 13;6:42. Originally published 2021 Feb 24. [Version 2] doi: 10.12688/wellcomeopenres.16168.2 (PMC8008441; doi:10.12688/wellcomeopenres.16168.2)
Supplement: Peer review history

# Peer review: Reviewer response for version 1

**DOI:** 10.21956/wellcomeopenres.17752.r42796

**Published:** 2021-03-25

**Authors:** Didier Menard

**Affiliations:** 1 Malaria Genetics and Resistance Unit, Parasites and Insect Vectors Department, Institut Pasteur, Paris, France

This manuscript from the MalariaGEN consortium, a data-sharing community of teams working on *Plasmodium falciparum* genomic epidemiology, presents the new release of curated *P. falciparum* genomes from isolates collected in 73 locations in Africa, Asia, South America and Oceania.

 Based on robust and perfectly detailed methods (ranging from the treatment of the blood samples, the DNA extraction, the Illumina and computational platforms developed to produce genome sequencing for variant discovery and genotype calling), they analyzed 7000 *P. falciparum* genome sequences and provided numerous exciting data. For instance, they found that variations (SNPs and indels) in *P. falciparum* genome affected about a quarter of the 23 Mb genome (and mostly coding regions), or that duplication genotypes are frequent around mdr1, plasmepsin2/3 and gch1, which are known to be associated with antimalarial drug resistance (including mefloquine, piperaquine and sulfadoxine/pyrimethamine).

 Moreover, population genetic analyses conducted on this largest available data resource, depict a comprehensive picture of *P. falciparum* parasite populations globally and sub populations at continental level. In the results, a large section is devoted to the description of the geographic patterns of validated molecular markers (SNPs and CNVs) associated with antimalarial drug resistance. By compiling data on all samples collected from 2002–2015, they present clear profiles of drug resistance by regional sub-populations for the most used antimalarial drugs. Finally, they reveal a global landscape regarding a major challenge for malaria elimination, that are deletions in hrp2 and 3 genes linked with false negative results of HRP2-based malaria RDT.

 Written in a very clear way, it must be point out that the authors have made huge efforts so that these data are understandable for a general audience, especially for the non-experts in genomics or for policy makers in malaria endemic countries. Their data effectively depict the main challenges currently encountered in the fight against malaria: the monitoring of the strategies deployed by the assessment of the impact on *P. falciparum* parasite populations, the geographical evolution of antimalarial drug resistances and the effectiveness of diagnostic tools used in malaria endemic areas (i.e. malaria RDT).

 Of note, the authors fairly expose the main issues and drawbacks related to the methods used (i.e. the analytical challenges due to long tracts of highly repetitive sequence and hypervariable regions within the *P. falciparum* genome, and the challenges of studying a complex mixture of genotypes from polyclonal infections),

 Although, I am impressed by the work done by the consortium, I have several minor comments that could improve the manuscript:

- Sample collection - *P. falciparum* samples investigated are not from systematic sampling collections dedicated to this study but rather from multiple studies conducted by groups with different objectives and from heterogeneous populations (patients living in malaria endemic areas, travelers, etc.) . I think this issue should be discussed in the manuscript.
- Likewise, the long time period covering the samples collection (2002–2015) is also a major bias which can alter the final results.
- I guess that all samples were collected from symptomatic patients seen at health facilities level? Unfortunately, this makes that data presented capture only *P. falciparum* populations infected this population. With the rise of new technologies, I am wondering whether the MalariaGEN consortium could investigate samples collected from asymptomatic individuals and explore the genomic profiles of this hidden reservoir but representing the major parasite biomass?
- I am aware that the authors have performed a difficult and complex exercise by providing high quality genomic data and comprehensive description of their data for a large audience. The major challenge that is not addressed in the manuscript is how these important data can be translated into concrete actions in the field by health providers.
- Last comment regarding the database. It will be helpful to provide for each sample/genome sequence, the location (country) and the date of collection.

Is the work clearly and accurately presented and does it cite the current literature?

Yes

If applicable, is the statistical analysis and its interpretation appropriate?

I cannot comment. A qualified statistician is required.

Are all the source data underlying the results available to ensure full reproducibility?

Yes

Is the study design appropriate and is the work technically sound?

Yes

Are the conclusions drawn adequately supported by the results?

Yes

Are sufficient details of methods and analysis provided to allow replication by others?

Yes

Reviewer Expertise:

Expert in antimalarial drug resistance

I confirm that I have read this submission and believe that I have an appropriate level of expertise to confirm that it is of an acceptable scientific standard.

# Response

**Published:** 2021-07-06

**Authors:** Richard Pearson

**Affiliations:** Wellcome Trust Sanger Institute, UK

We thank the reviewers for the extremely positive and supportive feedback. In their comments and suggestions both reviewers have well captured the spirit of this data resource and of the large collaborative network behind it. We are pleased to submit detailed responses and a revised version of the manuscript that addresses their comments.

***2.1) Sample collection - P. falciparum samples investigated are not from systematic sampling collections dedicated to this study but rather from multiple studies conducted by groups with different objectives and from heterogeneous populations (patients living in malaria endemic areas, travelers, etc.). I think this issue should be discussed in the manuscript.***

 Thanks for raising this point. On one hand, the heterogeneity of sampling approaches offers a unique opportunity to investigate questions in a variety of epidemiological settings in a systematic way. Specifics of each study are provided in ftp://ngs.sanger.ac.uk/production/malaria/pfcommunityproject/Pf6/Pf_6_partner_studies.pdf and users of the resource can contact individual investigators for further details. At the same time, we agree that this can also act as a confounder in some analysis, which is why we’ve devoted significant time to the curation of the dataset to make it “analysis ready”.

 As suggested, we have amended the manuscript in version 2 to include the considerations above in the paragraph: “Samples were collected by independent groups that were operative at a given time and in a given place with distinct objectives; while care needs to be taken when interpreting results spanning multiple years and geographical settings (e.g. aggregated trends of drug resistance prevalence), this heterogeneity also allows for the exploration of a wide range of epidemiological and transmission settings.”

***2.2) Likewise, the long time period covering the samples collection (2002–2015) is also a major bias which can alter the final results.***

 This is an important point in particular for interpreting drug resistance results, and one we explicitly bring out in the paragraph: “Note that samples were collected over a relatively long time period (2002–15) during which there were major changes in global patterns of drug resistance, and that the sampling locations represented in a given year depended on which partner studies were operative at the time. To alleviate this problem, we have also divided the data into samples collected before and after 2011 (Supplementary Data; Supplementary table 10), but temporal trends in aggregated data should be interpreted with due caution.”. Following the reviewer’s suggestion, we have now stressed this point further in our reply to point (2.1) above.

***2.3) I guess that all samples were collected from symptomatic patients seen at health facilities level? Unfortunately, this makes that data presented capture only P. falciparum populations infected this population. With the rise of new technologies, I am wondering whether the MalariaGEN consortium could investigate samples collected from asymptomatic individuals and explore the genomic profiles of this hidden reservoir but representing the major parasite biomass?***

 Asymptomatic infections are indeed an incredibly significant reservoir that needs to be explicitly considered to achieve a complete and accurate picture of the transmission landscape. The development of new technologies has begun to dig deeper and deeper in this area and initial results seem to be very encouraging that good quality data can indeed be obtained from asymptomatic and/or low parasitemia subjects. MalariaGEN would certainly be supportive of this kind of effort and we have indeed active collaborations with partners exploring these questions. To the best of our knowledge, though, some of these methodologies are still of limited sensitivity and in part experimental and will require further work in order to be deployed on the large scale required by this scientific question, but that is certainly an area for future investigation.

***2.4) I am aware that the authors have performed a difficult and complex exercise by providing high quality genomic data and comprehensive description of their data for a large audience. The major challenge that is not addressed in the manuscript is how these important data can be translated into concrete actions in the field by health providers.***

 This data resource represents a clear step towards the ultimate objective of translating genomic surveillance outputs into actionable actions, although it is fair to say that this is a long journey with many different components. The ability for multiple groups to share data, to analyse it using standardised methods, and to make it readily accessible is the foundation for translational impact to reach maturity.

 In the discussion we highlighted a series of future translational directions which have been and will be facilitated by resources like this one (and future ones) but it is certainly true that these results require careful interpretation due to the caveats highlighted in the paper and by the reviewer, which inevitably limit their impact. At the same time this dataset does create a systematic framework to enact and contextualize future discoveries of that nature and, indirectly, contributes to them.

 Ultimately, the practical value for malaria control will be greatly enhanced by the progressive acquisition of longitudinal time-series data and their integration with other sources of epidemiological data which will allow control programmes to monitor the impact of their interventions on the parasite population in near real time.

***2.5) Last comment regarding the database. It will be helpful to provide for each sample/genome sequence, the location (country) and the date of collection.***

 This information is included in the “Sample provenance and sequencing metadata” file available at the resource page https://www.malariagen.net/resource/26

# Peer review: Reviewer response for version 1

**DOI:** 10.21956/wellcomeopenres.17752.r42794

**Published:** 2021-03-22

**Authors:** Maria Isabel Veiga, Nuno S. Osório

**Affiliations:** 1 ICVS/3B's - PT Government Associate Laboratory, University of Minho, Braga, Portugal

The analysis of whole-genome sequences obtained from *Plasmodium falciparum* is particularly challenging due to the presence of hypervariable regions, highly repetitive sequences, and frequent mixture of parasites due to multiple infections of the host. The authors of this study describe a curated list of over three million high-confidence polymorphisms obtained from the genome sequence analysis of more than 7000 samples of *P. falciparum* collected by several studies in 73 locations in Africa, Asia, South America and Oceania.

 This work, reporting a laudable effort to substantially enrich publicly available genome data of *P. falciparum* worldwide, is of paramount importance for the field. The contribution goes in line with authors' previous consortia publications, extending largely the number of available data that can be analysed via web with powerful data analysis pipelines. By providing open access to a curated list of polymorphisms based on reproducible and high-quality protocols for the sequencing and analysis of *P. falciparum* genomes this study is likely to decrease the difficulties that have delayed the research on genomic epidemiology and population genomics of *P. falciparum*. Among other advances, studies in this area are likely to have important implications for a better understanding of the evolution towards drug resistance of the different global parasite populations ultimately contributing for a better control of this devastating disease. The manuscript is very well written and clear. It presents eight genetically distinct populations of parasites each endemic to different word regions, including South America, West Africa, Central Africa, East Africa, South Asia, West Southeast Asia, East Southeast Asia and Oceania. An interesting genetic and geographic characterization of the eight parasite populations is also shown. Of note, the finding of higher within-host diversity in the parasite populations endemic to Africa, the identification of single nucleotide polymorphism with high levels of geographic differentiation, and further characterization of geographic patterns of drug resistance and polymorphisms with potential impact in rapid diagnostic tests. We do not have major criticisms of the study.

**Our minor suggestions for the improval of the manuscript focus on:**

- Increasing the accessibility of the table listing polymorphisms in supplementary data. The authors do provide the data in VCF and zarr files, which are not very user friendly nor allow a fast search of a specific polymorphism. We understand that developing a web interface for this purpose would be a challenge beyond this research article but possibly exporting the VCF file data into tables that could be available in online repositories.
- Add to the supplementary file 4, describing the drug resistance markers genotype, the PfMDR1 N86Y. This SNP is a well-known modulator of antimalarial response and considered a risk factor for the treatment of artemether-lumefantrine.
- Add the ID of the genes most mentioned in the main article. The gene ID (PF3D7_xxxxxxx), is provided in supplementary file 7, but to clarify the reader, we recommend to add it also in the main article when first describing the genes.
- In the results section, when describing gene amplification and different sets of breakpoints, the authors describe complex rearrangements that have not been observed before in Plasmodium species. In regards to pfmdr1 duplication events has been described to vary in size while spanning different genes in different parasites ^1^^,^^2^^,^^3^^,^^4^. In a genome walking like approach, it has been described different amplicon sizes containing the pfmdr1 in clinical isolates from Southeast Asia where they also investigated if the type (i.e., which genes are included) and size of the amplicon influence drug susceptibility phenotypes ^5^.

Is the work clearly and accurately presented and does it cite the current literature?

Yes

If applicable, is the statistical analysis and its interpretation appropriate?

Yes

Are all the source data underlying the results available to ensure full reproducibility?

Yes

Is the study design appropriate and is the work technically sound?

Yes

Are the conclusions drawn adequately supported by the results?

Yes

Are sufficient details of methods and analysis provided to allow replication by others?

Yes

Reviewer Expertise:

Molecular epidemiology, antimalarial drug resistance

We confirm that we have read this submission and believe that we have an appropriate level of expertise to confirm that it is of an acceptable scientific standard.

## References

[1] : Amplification of the multidrug resistance gene in some chloroquine-resistant isolates of P. falciparum. *Cell* .1989;57(6) : 10.1016/0092-8674(89)90330-9 921-930 10.1016/0092-8674(89)90330-9 2701941

[2] : Recurrent gene amplification and soft selective sweeps during evolution of multidrug resistance in malaria parasites. *Mol Biol Evol* .2007;24(2) : 10.1093/molbev/msl185 562-73 10.1093/molbev/msl185 17124182

[3] : Amplification of the multidrug resistance gene pfmdr1 in Plasmodium falciparum has arisen as multiple independent events. *Mol Cell Biol* .1991;11(10) : 10.1128/mcb.11.10.5244 5244-50 10.1128/mcb.11.10.5244 1922044PMC361573

[4] : Genome wide gene amplifications and deletions in Plasmodium falciparum. *Mol Biochem Parasitol* .2007;155(1) : 10.1016/j.molbiopara.2007.05.005 33-44 10.1016/j.molbiopara.2007.05.005 17599553

[5] : pfmdr1 amplification is related to increased Plasmodium falciparum in vitro sensitivity to the bisquinoline piperaquine. *Antimicrob Agents Chemother* .2012;56(7) : 10.1128/AAC.06350-11 3615-9 10.1128/AAC.06350-11 22508315PMC3393437

# Response

**Published:** 2021-07-06

**Authors:** Richard Pearson

**Affiliations:** Wellcome Trust Sanger Institute, UK

We thank the reviewers for the extremely positive and supportive feedback. In their comments and suggestions both reviewers have well captured the spirit of this data resource and of the large collaborative network behind it. We are pleased to submit detailed responses and a revised version of the manuscript that addresses their comments.

***1.1) Increasing the accessibility of the table listing polymorphisms in supplementary data. The authors do provide the data in VCF and zarr files, which are not very user friendly nor allow a fast search of a specific polymorphism. We understand that developing a web interface for this purpose would be a challenge beyond this research article but possibly exporting the VCF file data into tables that could be available in online repositories.***

 We thank the reviewer for this important feedback on how to increase the reach of this resource. Since the publication of this article, we have been working on an initial web interface that allows users to navigate some aspects of the data: please see https://www.malariagen.net/apps/pf6. The current version mainly focuses on epidemiologically relevant data and emphasises the community behind the project and at the moment doesn’t provide access to the genomic variation information, which will require further work.

 Of course accessibility is a relative criteria and as such it requires balancing out different priorities. In the past we have provided tabular versions of the data ( www.malariagen.net/data) but the benefits have been very limited. For example, handling multiallelic and non-SNP variations requires somewhat arbitrary encoding decisions that significantly affect the simplicity and intuitiveness of the tabular format. Increasing the sample size has made these variations more common (e.g. in this release there are about 50% non-SNP variants and 50% multiallelic variants) to the point that there was no real advantage in maintaining the format. The decision of primarily utilising the VCF format comes from the recognition that these files are the standard de facto in the genomic community, which in turn has developed a large ecosystem of tools to handle them: please see the README at ftp://ngs.sanger.ac.uk/production/malaria/pfcommunityproject/Pf6/Pf_6_README_20191010.txt for some examples, e.g. to subset the data.

 However we agree this might still be limiting for some use cases and we are working towards a more integrated solution. As an example of our direction of travel, please see https://malariagen.github.io/vector-data/landing-page.html, which presents some simplified data access workflows for the MalariaGEN *Anopheles gambiae* 1000 Genomes Project.

***1.2) Add to the supplementary file 4, describing the drug resistance markers genotype, the PfMDR1 N86Y. This SNP is a well-known modulator of antimalarial response and considered a risk factor for the treatment of artemether-lumefantrine.***

 We recognise that there is growing evidence of the role of *PfMDR1* N86Y in artemether-lumefantrine resistance. In particular, multiple studies have shown that lumefantrine appears to select for N86. Despite that, WHO still reports markers of resistance to lumefantrine as “Yet to be validated” (p. 22 - **https://www.who.int/publications/i/item/9789240012813**). In this release, supplementary file 4 only contains validated markers so it would be inconsistent to add the markers. However, we will consider adding putative markers in future releases where appropriate.

***1.3) Add the ID of the genes most mentioned in the main article. The gene ID (PF3D7_xxxxxxx), is provided in supplementary file 7, but to clarify the reader, we recommend to add it also in the main article when first describing the genes.***

 We have implemented the recommendation and added gene IDs every time a gene is mentioned for the first time in the manuscript version 2.

***1.4) In the results section, when describing gene amplification and different sets of breakpoints, the authors describe complex rearrangements that have not been observed before in Plasmodium species. In regards to pfmdr1 duplication events has been described to vary in size while spanning different genes in different parasites1,2,3,4. In a genome walking like approach, it has been described different amplicon sizes containing the pfmdr1 in clinical isolates from Southeast Asia where they also investigated if the type (i.e., which genes are included) and size of the amplicon influence drug susceptibility phenotypes5.***

 The complex rearrangements that have not been observed before which we were referring to here are “dup-trpinv-dup” rearrangements that to the best of our knowledge have only previously been described in human data (see ref 58). This complex and large structural rearrangement involves a triplicated segment embedded within a duplication, in which the triplicated segment is inverted. We recognise that the original wording in the text was ambiguous and we’ve replaced “complex rearrangements” with an explicit description of the event.
